# An extended OpenSim knee model for analysis of strains of connective tissues

**DOI:** 10.1186/s12938-018-0474-8

**Published:** 2018-04-17

**Authors:** M. Marieswaran, Arnab Sikidar, Anu Goel, Deepak Joshi, Dinesh Kalyanasundaram

**Affiliations:** 1 Centre for Biomedical Engineering, Indian Institute of Technology Delhi, New Delhi, 110016 India; 2 Department of Biomedical Engineering, All India Institute of Medical Sciences, New Delhi, 110029 India

**Keywords:** Musculoskeletal model, OpenSim, Ligament, Strain, Differential intra-bundle strain

## Abstract

**Background:**

OpenSim musculoskeletal models provide an accurate simulation environment that eases limitations of in vivo and in vitro studies. In this work, a biomechanical knee model was formulated with femoral articular cartilages and menisci along with 25 connective tissue bundles representing ligaments and capsules. The strain patterns of the connective tissues in the presence of femoral articular cartilage and menisci in the OpenSim knee model was probed in a first of its kind study.

**Methods:**

The effect of knee flexion (0°–120°), knee rotation (− 40° to 30°) and knee adduction (− 15° to 15°) on the anterior cruciate, posterior cruciate, medial collateral, lateral collateral ligaments and other connective tissues were studied by passive simulation. Further, a new parameter for assessment of strain namely, the *differential inter*-*bundle strain* of the connective tissues were analyzed to provide new insights for injury kinematics.

**Results:**

ACL, PCL, LCL and PL was observed to follow a parabolic strain pattern during flexion while MCL represented linear strain patterns. All connective tissues showed non-symmetric parabolic strain variation during rotation. During adduction, the strain variation was linear for the knee bundles except for FL, PFL and TL.

**Conclusions:**

Strains higher than 0.1 were observed in most of the bundles during lateral rotation followed by abduction, medial rotation and adduction. In the case of flexion, highest strains were observed in aACL and aPCL. A combination of strains at a flexion of 0° with medial rotation of 30° or a flexion of 80° with rotation of 30° are evaluated as rupture-prone kinematics.

**Electronic supplementary material:**

The online version of this article (10.1186/s12938-018-0474-8) contains supplementary material, which is available to authorized users.

## Background

The human knee is a critical load bearing joint that experiences 200–300% of the body weight during various kinetic activities such as standing up, sitting down, standing on one foot, level walking, ascending stairs, descending stairs etc., [1–3]. Multiple ligaments and tendons hold the complex knee joint together. Studies suggest that the rate of ligament injuries are considerably higher in athletes (~ 54%) compared to general day-to-day activities (~ 36%) and trauma (< 12%) [4–7]. Of the several ligaments in the knee joint, anterior cruciate ligament (ACL) encounters the highest frequency of injuries during dynamic events and therefore the study of ACL injury in sports is a well-researched topic of interest [8]. Majority of the published studies on ACL injuries have explored the diagnosis and surgical treatment, post-surgical rehabilitation programs, procedures to facilitate speedy recovery and post-injury biomechanics [9–12]. A handful number of cadaveric studies were reported on mechanical and structural properties of knee ligaments [13, 14]. Though the information provided by these studies are quite valuable, there are limitations such as differences in sources of cadaveric tissues, preservation modes of tissues, in vitro conditions, experimental design and loading parameters etc., [15]. However, the mechanical behaviour of ACL and other ligaments in-line with real-life kinematics and loading conditions shall help us in a better understanding of the stress–strain relationship, injury mechanisms etc.

In the past, various methods were employed to study the strain behaviour of the ligaments and tendons. The use of surface electromyogram (sEMG) during dynamic sports activities was one of the approaches. However, dynamic motion during sports offers jerk over the sensor results in spatial re-orientation of the sensor. Further, sEMG sensors create hindrance towards the natural motion of the subject during sports activities [16–19]. Studies involving in vivo testing using surgically implanted measurement devices have also been used to estimate ACL tension during human walking and other activities [13, 15, 20]. However, such an approach is invasive and introduces additional complexities. The study of strain on cadaveric ligaments and tendons is an alternate method for assessment of strain, however, factors such as availability of samples, ethical clearances etc., limit the kinematic studies [19, 21–25]. Hence, in order to understand strains of the connective tissue, there is a need for musculoskeletal models in simulation-based studies.

Among various musculoskeletal models, a finite element knee model offers accurate analyses. However, the analysis is complex and computationally time-consuming [26]. OpenSim provides discrete element models that offer a balance of low computational time and a relatively complex anatomically correct model [10, 27]. Predicting muscle and soft tissue behaviour during gait using musculoskeletal models has been an area of research interest for the past few years [13, 27, 28]. Xu et al. [14] developed an OpenSim Gait model that includes four knee ligaments with 6 degrees of freedom (DOF) to the knee joint. Schmitz et al. [10] further developed Xu’s model to include articular cartilage, tibial plateaus and 18 bundles of connecting tissues. The authors substantiated the findings with cadaveric studies on specimens for structural properties [11]. However, menisci were absent in both the models. Menisci is a soft tissue that plays an important role in modifying the point contact of femoral cartilage with tibia into a uniform surface contact by facilitating a larger area of contact that decreases the contact pressure [29]. The inclusion of menisci leads to realistic strains in the connective tissues. In summary, the development of an OpenSim musculoskeletal gait model with a serial joint knee comprising all ligaments, capsules, menisci and femoral articular cartilages to an anatomically correct system is presented here. The strains in the connective tissue bundles are evaluated under three rotational kinematics of the knee.

## Methods

**Fig. 1:**
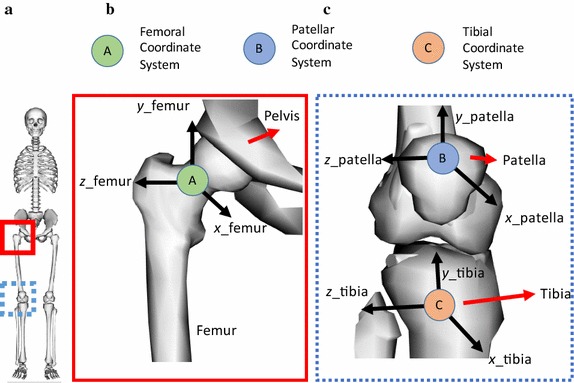
**a** Human skeletal system; **b** reference frame of femur coordinate system; **c** reference frames for patella and tibia

A base musculoskeletal model of Xu et al. was adopted from https://simtk.org/home/kneemodel/. It was further developed by incorporating menisci, articular cartilages that tallied with the boundary femoral condyle, transverse ligament (TL), menisco-femoral ligaments (MFL), patella-tibial ligament (PT), capsules (CAP), popliteofibular ligament (PL), fibular ligaments (FL), and patello-femoral ligaments (PFL) [10, 11]. The 3D models for cartilages and menisci were assimilated from a volunteer’s magnetic resonance image (MRI) data through Mimics^®^ (Version 17.0, Materialise, Belgium). The detailed protocol of obtaining soft tissue (cartilage or menisci model) from MRI data is given in the Additional file 1. The ethical clearance was approved vide document number IEC/637 dated November 03, 2017. The knee joint consists of two articulations: (i) *tibio*-*femoral* articulation, wherein the medial and lateral condyles of the femur articulate against the tibia and (ii) *patellofemoral* articulation wherein the patella articulates against the patellar surface located between the lateral and medial groove of the femoral condyles. The articulations are discussed in the Additional file 1. Figure 1 depicts the coordinate frames of the articulation. The global coordinates of various entities are given in Additional file 1: Table S1.

### Tibio-femoral geometry and connective tissues

**Fig. 2:**
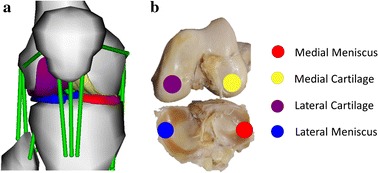
**a** Proposed model post inclusion of menisci; **b** a human cadaveric model

The geometry was adapted from an open-source finite element knee model of the right human knee [26]. This geometry was in accord with a 77.5 kg female subject and thus has to be scaled in order to approximate the anthropometry of Schmitz’s Model [10, 11, 13]. This scaling was performed to accommodate the geometry of right femoral articular cartilages in accordance with the condyles of the femur in the proposed model using Geomagic^®^ Wrap (Version 2015.1.3, 3D Systems, Inc.,) software. The OpenSim Model of the right knee joint is shown in Fig. 2. The knee joint contains six degrees of freedom (DOF) with three rotation and three translational movements. The three rotational DOFs such as knee rotation, knee adduction and medial–lateral translation were independent while anterior–posterior translation and proximodistal translation were a function of knee flexion–extension [14]. The detailed procedure to extract the geometry of the menisci and the articulations of the knee are provided in the Additional file 1.

**Fig. 3:**
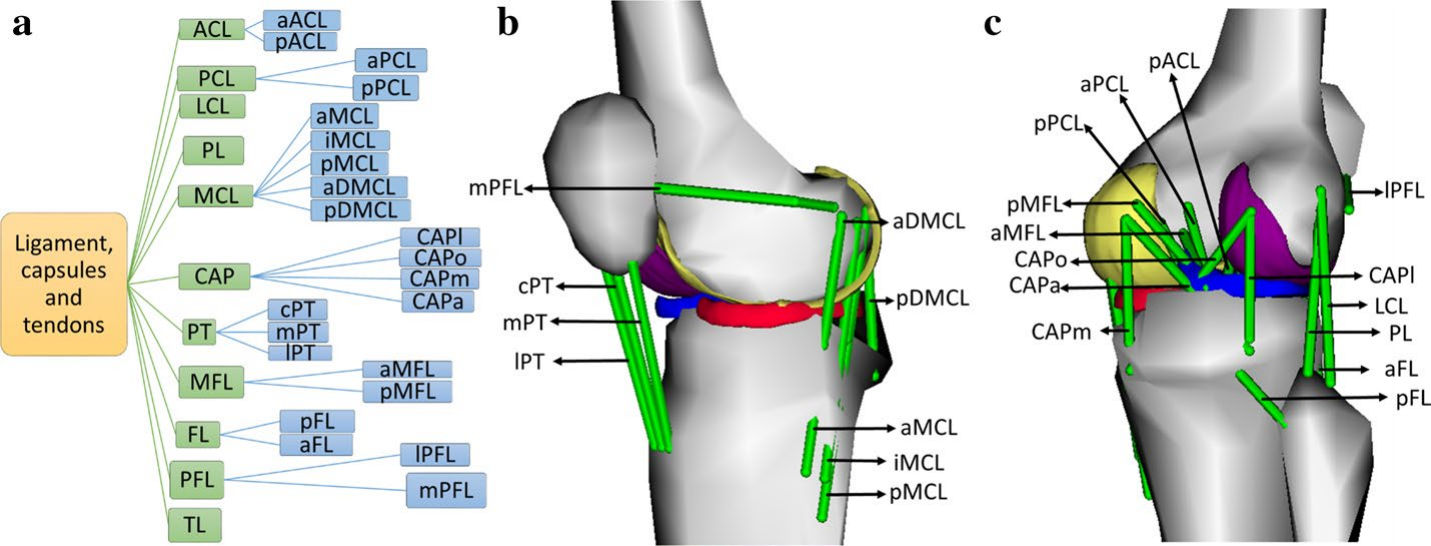
**a** Diagrammatic representation of 25 bundle fibres to represent connecting tissues; **b** anteromedial sagittal view; **c** posterior view

All the ligaments and tendons of the knee joint were modelled via 25 ligament bundles. The name of the tissues are as follows along with respective abbreviations and number of bundles in parenthesis: anterior cruciate ligament (ACL, 2), posterior cruciate ligament (PCL, 2), medial collateral ligament (MCL, 5), lateral collateral ligament (LCL, 1), popliteofibular ligament (PL, 1), posterior capsule (CAP, 4), patellar-tibia ligament (PT, 3), transverse ligament (TL, 1), menisco-femoral ligament (MFL, 2), medial patello-femoral ligaments (mPFL, 1), lateral patella-femoral ligament (lPFL, 1) and fibular ligament (FL, 2). The terminology of each of the 25 bundles are given in Fig. 3a. It should be noted that few researchers refer the patella-tibial ligament [30] as patellar tendon [31]. The position of each of the bundles on the anterior and posterior are shown in Fig. 3b, c respectively. The bundles were modelled as linearly elastic soft tissue. The initial tibial and femoral attachment sites of all the bundles used in this model were adapted from a previous study [14] and are given in Additional file 1: Tables S1 and S2 for easy reference. Ligament length is considered as the straight-line distance between femoral and tibial attachment sites. The resting length, resting strain and force at unit elongation are given in Additional file 1: Table S3 and Figure S2. The detailed protocol for obtaining menisci from MRI data is provided in the Additional file 1.

### Passive simulations of the knee under flexion, rotation and adduction

**Fig. 4:**
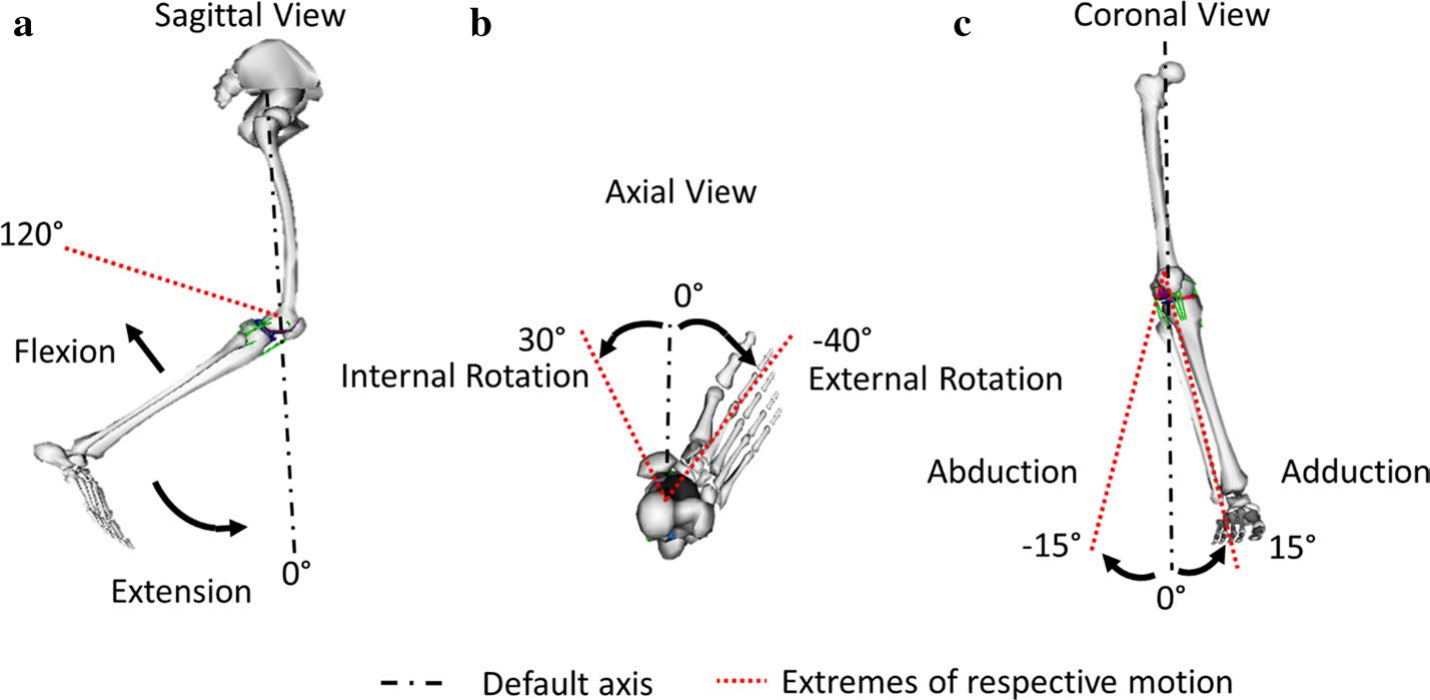
Knee joint under (**a**) flexion (**b**) rotation and (**c**) adduction

Three separate kinematics were evaluated under passive simulation viz. (a) flexion from 0° to 120°, (b) rotation from 40° externally to 30° internally and (c) adduction from − 15° to 15°. For all the three cases, the default position of the knee was taken at complete extension i.e. flexion, rotation and adduction angles assigned to 0°. It is to be noted that, during analysis of a particular case, other DOFs remain constrained. The flexion and extension, internal and external rotation and abduction and adduction of the knee are shown in Fig. 4a–c. Engineering strain is calculated as per Eq. (1).

1 $$Strain = \frac{{L_{f} - L_{0} }}{{L_{0} }}$$

where *L*_*f*_ is the extension and *L*_0_ is the resting length of the connective tissue.

Further, the strains between different bundles within the same connective tissue are calculated as *differential intra*-*bundle strain* (DIBS). The intra-ligament bundles are enclosed by a membrane constraining the bundles as a single unit [32, 33]. Therefore, the *differential intra*-*bundle strain* is significant to evaluate potential damage situations. Hence, DIBS and ultimate strain are two parameters that define the failure of the ligament. The equation for DIBS is given in Eq. (2).

2 $$DIBS_{i,j} = \left\{ {\begin{array}{*{20}l} {\left| {s_{i} - s_{j} } \right|, \quad s_{i} ,s_{j} > 0} \\ {0,\quad s_{i} ,s_{j} \le 0} \\ {\left\{ {\begin{array}{*{20}l} {0, (s_{i} + s_{j} ) \le 0} \\ {\left| {s_{i} + s_{j} } \right|, (s_{i} + s_{j} ) > 0)} \\ \end{array} } \right\}, \quad \forall (s_{i} < 0)\mathop \cap \nolimits (s_{j} > 0)} \\ \end{array} } \right.$$

where *s*_*i*_ = strain in *i*th bundle, *s*_*j*_ = strain in *j*th bundle.

## Results

**Fig. 5:**
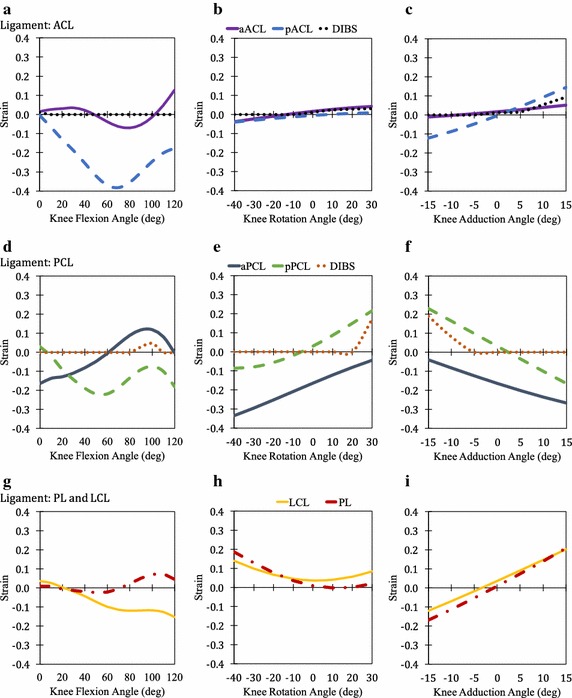
Passive strain behaviour of ACL (**a**, **b**, **c**), PCL (**d**, **e**, **f**), LCL, and PL (**g**, **h**, **i**)

**Fig. 6:**
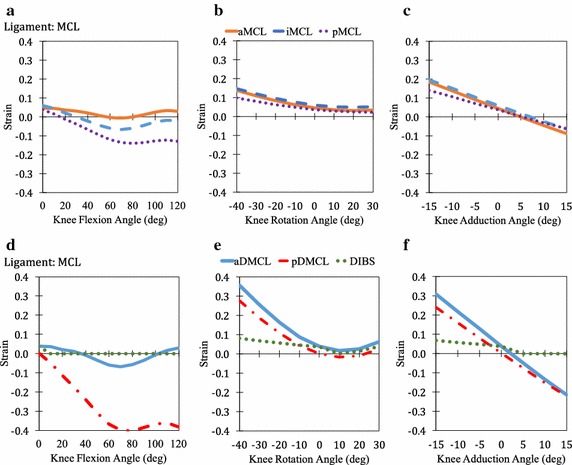
Passive strain behaviour of MCL (**a**, **b**, **c**, **d**, **e**, **f**)

**Fig. 7:**
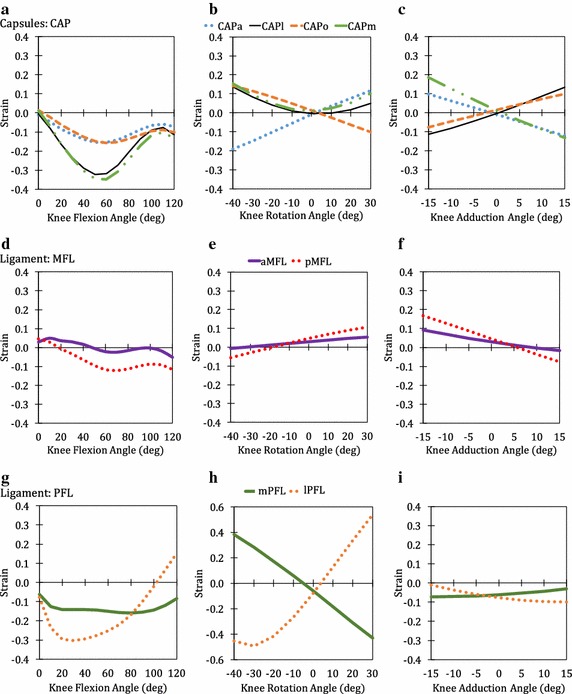
Passive strain behaviour of CAP (**a**, **b**, **c**), MFL (**d**, **e**, **f**) and PFL (**g**, **h**, **i**)

The strains of twenty-five bundles of the knee joint were studied at three different independent angular kinematics. The strain is calculated as per Eq. (1). The results of the study are shown in Figs. 5, 6 and 7 except for 6 of the bundles which does not show significant strains or change in strains. The strain plots of aFL, pFL, cPT, mPT, lPT and TL during flexion, rotation and adduction are shown in Additional file 1: Figure S3 for reference.

During flexion, the strain variations of ACL, PL, MCL, CAP, MFL, and PFL was observed to follow a partially parabolic pattern i.e. decrease in strain initially followed by increase in strain at higher flexion angles. In the case of aPCL, an inverse partial parabolic pattern was noticed while a partial parabolic pattern was observed in pPCL. No change from resting strain was observed for FL and TL. An approximately linear pattern was observed in the case of PT during flexion as well as in the other two kinematics. During rotation, the strain variation in many of the bundles followed a non-symmetric parabolic contour in the case of pPCL, LCL, PL, MCL (all five bundles), CAPl, CAPm and lPFL. For the other bundles, aACL, pACL, aPCL, CAPa, CAPo, aMFL, pMFL and mPFL linear variation in strain was observed. No change from resting strain was observed for aFL, pFL, cPT, mPT, lPT and TL. During adduction, the strain variation was linear or constant for all the twenty-five knee bundles. Linear variation with positive slope indicates higher strains during adduction while negative slope indicates higher strains during abduction. The maximum value of the strains of each of the bundles and its corresponding angles of flexion, rotation and adduction are given in Table 1 for easy reference. The maximum value of DIBS in each of the connective tissues are given in Table 2.

**Table 1:** Maximum strain of connective tissue bundles

Connective tissue	Bundle	Maximum strain		
		Flexion (@angle)	Rotation (@angle)	Adduction (@angle)
ACL	aACL	0.128 (120°)	0.041 (30°)	0.051 (15°)
	pACL	− 0.004 (0°)	0.010 (30°)	0.144 (15°)
PCL	aPCL	0.120 (100°)	− 0.044 (30°)	− 0.040 (− 15°)
	pPCL	0.030 (0°)	0.216 (30°)	0.228 (− 15°)
LCL	LCL	0.036 (0°)	0.139 (− 40°)	0.202 (15°)
PL	PL	0.073 (110°)	0.188 (− 40°)	0.208 (15°)
MCL	aMCL	0.046 (0°)	0.138 (− 40°)	0.184 (− 15°)
	iMCL	0.061 (0°)	0.148 (− 40°)	0.195 (− 15°)
	pMCL	0.037 (0°)	0.099 (− 40°)	0.141 (− 15°)
	aDMCL	0.037 (0°)	0.357 (− 40°)	0.309 (− 15°)
	pDMCL	0.001 (0°)	0.275 (− 40°)	0.240 (− 15°)
CAP	CAPa	− 0.009 (0°)	0.116 (30°)	0.097 (− 15°)
	CAPl	− 0.004 (0°)	0.136 (− 40°)	0.134 (15°)
	CAPo	0.014 (0°)	0.143 (− 40°)	0.099 (15°)
	CAPm	0.012 (0°)	0.154 (− 40°)	0.185 (− 15°)
MFL	aMFL	0.049 (10°)	0.054 (30°)	0.092 (− 15°)
	pMFL	0.046 (0°)	0.106 (30°)	0.167 (-15°)
PFL	mPFL	− 0.085 (120°)	0.383 (− 40°)	− 0.031 (15°)
	lPFL	0.154 (120°)	0.538 (30°)	− 0.010 (− 15°)
PT	cPT	0.028 (120°)	− 0.046 (constant)	− 0.046 (constant)
	mPT	0.102 (120°)	0.030 (constant)	0.030 (constant)
	lPT	0.103 (120°)	0.030 (constant)	0.030 (constant)

**Table 2:** Maximum DIBS of connective tissue

Connective tissue	DIBS		
	Flexion (@angle)	Rotation (@angle)	Adduction (@angle)
ACL	0.01 (0°)	0.03 (30°)	0.09 (15°)
PCL	0.05 (100°)	0.17 (30°)	0.19 (− 15°)
LCL	Not applicable		
PL	Not applicable		
MCL	0.04 (0°) (aDMCL–pDMCL)	0.08 (− 40°) (aDMCL–pDMCL)	0.07 (− 15°) (aDMCL–pDMCL)
CAP	Not applicable		
MFL	Not applicable		
PT	0.075 (100°) (cPT–lPT)	0.08 (cPT–lPT) constant	
FL	Not applicable		
PFL	Not applicable		
TL	Not applicable		

## Discussion

The importance of realistic knee model is necessary for the study of ligament kinematics and dynamics. The model can serve as an alternative to cadaveric studies due to complexities involved, for example, low availability of samples, storage requirements and extensive time for preparation etc., [34, 35]. A finite element model eludes the complexities associated with cadaveric studies however, it suffers in mimicking the complex organic geometries of the human anatomical structure. In order to understand the complex mechanism of connective tissue injury, a more pragmatic model is desired. Discrete element OpenSim models are a bridge between both the extremes and hence was chosen for the present study. The proposed model includes all 25 connecting bundles, femoral articular cartilages and menisci to facilitate a realistic model of the knee joint. The model was passively simulated in the three independent rotations to study the strains on individual connective tissues. Mechanism of sports injuries [8, 21, 22, 36] and injuries in elderly [5, 15, 28] are lucidly credible with the help of the proposed model.

### Strains in the connective tissues

In the case of flexion, most bundles are not strained above 0.1 except for aACL, aPCL and lPFL. The highest number of the knee bundles for which strains exceeded 0.1 (Table 1) occurred during lateral rotation followed by abduction, medial rotation and adduction. These kinematics wherein the bundles strained over 0.1 can be quite dangerous leading to rupture of the bundle eventually. During lateral rotation, the bundles that were highly strained during are LCL, PL, MCL (all five bundles), CAPl, CAPo, CAPm and mPFL. These bundles are either placed anatomically in vertical orientation or at perfectly horizontal orientation resisting the rotation (Fig. 3b, c). Hence, high strains were observed. In addition, the strain pattern of these bundles were observed to be parabolic in nature.

In the case of adduction, the strains were found to linearly vary as a function of angle. However, high strains were observed in both abduction and adduction. It was noted that the bundles attached at the posterior part and medial part of the knee shows maximum strains at 15° of lateral rotation except for CAPl, CAPo, CAPm and lPFL. It is to note that the absolute values of a strain of the connective tissue depend upon the initial values of resting strains considered from the references. The values of the strains are bound to change depending upon the resting strains. However, the strain pattern with respect to the angle of flexion, rotation and adduction will remain unchanged at different resting strains.

### Combinational strains in kinematics

For ACL (Fig. 5a–c), a flexion of 120° results in a strain of ~ 0.13 which is near to the ultimate strain for ACL (~ 0.15) [37]. This kinematics imposes maximum strain in the aACL and therefore, any other kinematics accompanied with 120° of flexion may intigate failure in ACL. Abduction of 15° in pPCL (Fig. 5d–f), will result in a strain of ~ 0.23 which is equal to the ultimate strain for PCL (~ 0.23) [38]. A medial rotation of tibia at 30° with 0° flexion adds up to a strain of ~ 0.24 in pPCL which exceeds the ultimate strain for PCL. This kinematics is commonly witnessed during sports injuries of football [39] and may instigate rupture. In case of LCL (Fig. 5g–i), complete adduction or complete lateral rotation of knee exceeds its ultimate strain (~ 0.10) [40] from cadaveric data and therefore is a failure prone kinematics for the ligament. These kinematics are commonly witnessed in sports such as American football and Indian kabaddi [8, 41]. At 20° lateral rotation, the strain in aDMCL and pDMCL (Fig. 6e) is greater than the ultimate strain for MCL i.e. ~ 0.13 [40]. However, abduction of distant MCLs at their extremes may result in a failure-prone kinematics as it exceeds the ultimate strain for MCL [40]. Sports actions involving physical tackle for instances English rugby or American football or Indian kabbadi causes similar kinematics, leading to rupture of MCL [42].

### DIBS

As described earlier, DIBS is a parameter to analyse the strain in the two bundles that are anatomically enclosed by a synovial membrane such as aACL and pACL [32]. Table 2 illustrates DIBS for ACL, PCL, MCL and PT. DIBS is not applicable for MFL, PFL and CAP as the bundles are not anatomically enclosed within a membrane [43]. As there is no experimentally defined upper limit of DIBS to be considered dangerous, a value of 0.1 is considered as the rupture-prone limit for the sake of discussion. In ACL, maximum DIBS of 0.09 was observed at 15° adduction which is close to the ultimate strain of ACL (~ 0.15) [37] and therefore, may induce high strain to the ligament. For PCL, the maximum DIBS of 0.19 was observed which is close to the ultimate strain of PCL (~ 0.23) [38] and therefore can induce high strain in the ligament. In MCL, the maximum DIBS was observed during rotation i.e. ~ 0.08 which close to the ultimate strain of MCL (~ 0.13) [40]. This type of kinematics strains the ligament to its maximum and can instigate rupture on implication of external loading. During rotation and adduction, nearly constant strains with DIBS less than 0.1 was observed throughout the range of angles on the patellar tendons. DIBS is one of the parameters to evaluate the damage to the ligament while the other being the ultimate strain of the ligament.

## Conclusions

The following are the summarized points from the discussion on the strains of the connective tissue bundles of the knee joint:

- During flexion, the strain variation of ACL, PL, MCL, CAP, MFL, and PFL was observed to follow a partial parabolic pattern i.e. decrease in strain initially followed by increase in strain at higher flexion angles. In the case of LCL, a non-linear decreasing strain pattern was observed. An inverse semi-parabolic strain pattern was observed in the case of aPCL. No significant change in strain was observed for FL, PT and TL.
- During rotation, the strain variation in many of the bundles followed a non-symmetric parabolic contour except in the case of CAPa, CAPo, aMFL, pMFL, mPFL and lPFL, where the strain variation was linear. No change from resting strain was observed for FL, PT and TL. The parabolic pattern of strain characteristics was observed for the connective tissues that are anatomically placed in a vertical or near vertical direction between femur and tibia such as collateral ligaments and cruciate ligaments. Therefore, the maximum strain was observed at either of the two extremes of medial and lateral rotations or at both the extremes depending on the offset from the centre of rotation.
- During adduction, the strain variation was linear for the knee bundles except for FL, PT and TL. Linear variation with positive slope indicates higher strains during adduction while negative slope indicates higher strains during the abduction. All connecting tissues show linear strain characteristics, again vowing to the vertical alignment.
- The tissues were assumed to be linearly elastic in this study, which is a limitation. However, the tissues in real-life exhibit non-linear visco-elasticity.
- Complete characterization of the ligament failure needs consideration of both ultimate strain and DIBS. The significance of DIBS is to indicate a potential shear between anatomically connected bundles.

The application of this model lies in the study of various mechanisms of knee injury by simulating it with various sports activities. Moreover, the customizability of the model aids in the incorporation of fixtures, dampers, prosthetics and orthotic devices etc. under simulation with the prediction of various trauma mechanisms and remedies.

## Additional file

**Additional file 1.** Modeling and articulation.
