# Supplementary material for: An extended OpenSim knee model for analysis of strains of connective tissues
Source: Biomed Eng Online. 2018 Apr 17;17:42. doi: 10.1186/s12938-018-0474-8 (PMC5905155; doi:10.1186/s12938-018-0474-8)
Supplement: Supplementary file 1 — Additional file 1. Modeling and articulation. [file 12938_2018_474_MOESM1_ESM.docx]

**A novel parameter to assess the strains of ligaments and tendons in an extended OpenSim knee model**

**Additional file**

**Procedure to extract soft tissues from MRI Data**

The MRI data or DICOM data is imported into Mimics® (Version 17.0, Materialise, Belgium) which converts the image data into 3D model using image segmentation techniques. The soft tissues from MRI data are extractred using the region growing tool of Mimics where the homoginity criteria is set by the number of connectivity points. 26 connective points are used to calculate the seed points for region growing. Once the operation is carried out, the mask is further threshold to extract the abstract mask. The masking toolbox is used for refining the mask. After extracting the refined mask, the mask was used for converting the images to stereolithography (STL) file i.e. the 3D geometrical model. The 3D geometrical model is further refined using filteration, smoothening and triangle removal operations to attain the finished 3D model.


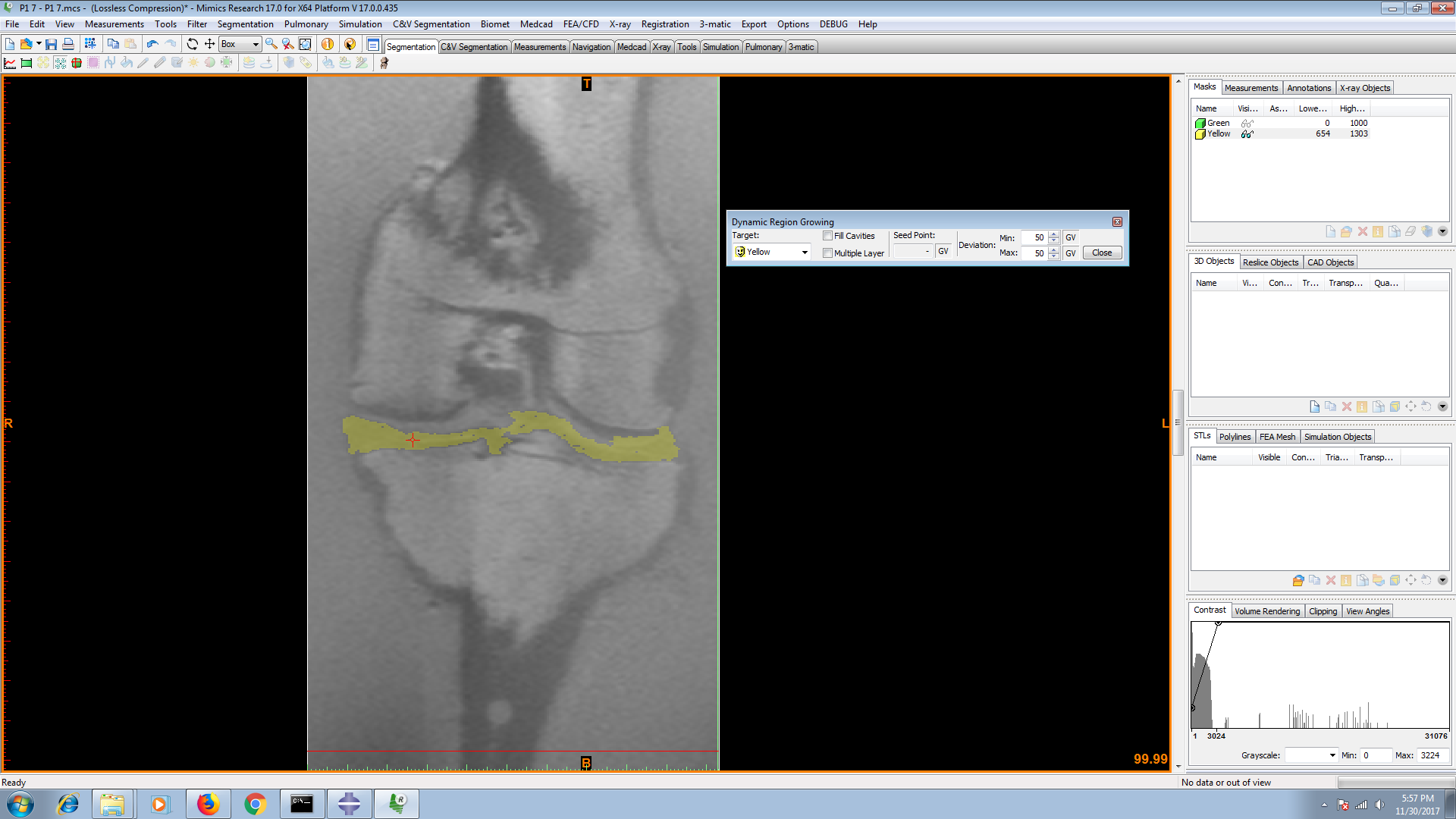


Figure S1. Dynamic region growing in Mimics masking the menisci.

## Tibio-femoral articulation

The articulation refers to the kinematic relationship between the femoral frame and the tibial frame. The centre of the knee is located at the transverse axis passing through the centre of the intercondyloid fossa. The origin of the tibial reference frame was located at the centre of intercondylar eminence. The positive *z*-axis was fixed collinear with the transverse axis passing through the intercondyloid fossa and pointing outwards laterally. The positive *y*-axis axis was considered perpendicular to the *z*-axis and pointing towards the femur. The positive *x*-axis was implicated perpendicular to both the axes pointing anteriorly. The femoral reference frame follows the same orientation as that of the tibial frame at the trochanteric chest of the proximal femur. Six degrees of freedoms (DOFs) with three rotations and three translations were included. Abduction-adduction, internal-external rotations, and knee flexion-extension are the rotational DOFs about *x*-axis, *y*-axis and *z*-axis respectively. Similarly, the translational DOFs consists of anterior-posterior translations, proximal-distal translations, medial-lateral translations along the *x*-axis, *y*-axis and *z*-axis respectively. The knee joint contains six degrees of freedom (DOF) with three rotation and three translational movements. The three rotational DOF along with mediolateral translation were independent while anterior-posterior translation and proximodistal translation were a function of knee flexion-extension. The other independent DOF are knee rotation, knee adduction and medial-lateral translation. A similar but separate coordinate system is defined for the left knee, however; the passive simulation was carried out on the right knee assuming uniformity between the two knees.

## Patello-femoral articulation

This articulation depicts the kinematic relationship between the femoral and patellar frame. The patellar coordinate system was oriented similarly to the femoral coordinate system. The origin was placed at the patellar surface located between the lateral and medial groove of the femoral condyles under a complete extension of the knee. Similar to models in the literature [1] one DOF was defined for this articulation, where patella is moved in a constrained path about distal femur [2]. The constrained path along this DOF is a linear function of *x*-axis translation along with *z*-axis rotation of the tibia [2]. These translations are expressed in the femoral coordinate system and defined with respect to patellar origin [2].

The articulations were constrained through muscle fibres defined under the Thelen 2003 muscle class [3] incorporating the properties of muscles from literature

## Inclusion of menisci

The meniscus is a fibrocartilage located on the proximal end of the tibial bone that aids in re-distributing the load on the knee joint. Menisci establish a larger area of contact with the articulating femoral cartilage, thanks to its wedge shape. The wedge shape also helps the femoral cartilage to roll /slide over it. Given its soft tissue characteristics, the fibrocartilage also absorbs the shocks originating from either end. To include menisci, MRI data of knee joint was imported in Mimics ®. The upper threshold for mask detection was set manually to 996 Hounsfield unit to extract the soft tissues from the data. Unwanted soft tissues were edited manually [4]. Post-processing like smoothening (factor 0.8) and triangular error removal (factor of 0.6) were carried out to refine the menisci. Finally, the menisci were tessellated into stereolithography (STL) format. The menisci were scaled to fit the existing model. The position and orientation vectors for the menisci were fixed to establish surface contact with femoral cartilages.

**Table S1. Properties of the ligament, capsules and tendon bundles**

| Bundle | Resting length (m) | Reference for resting length | Force at unit elongation (N) | Reference |
| --- | --- | --- | --- | --- |
| aACL | 0.032 | [5] | 1500 | [5] |
| pACL | 0.025 |  | 1600 |  |
| aPCL | 0.033 |  | 2600 |  |
| pPCL | 0.025 |  | 1900 |  |
| LCL | 0.055 |  | 2000 |  |
| aMCL | 0.070 |  | 2500 |  |
| iMCL | 0.069 |  | 3000 |  |
| pMCL | 0.085 |  | 2500 |  |
| aDMCL | 0.036 |  | 2000 |  |
| pDMCL | 0.037 |  | 4500 |  |
| PFL | 0.046 |  | 1620 |  |
| TL | 0.014 | [6] | 1020 | [7] |
| aFL | 0.020 | [8] | 1034 | [9] |
| pFL | 0.020 |  | 644 |  |
| aMFL | 0.027 | [10] | 810 | [11] |
| pMFL | 0.031 |  | 936.2 |  |
| CAPa | 0.056 | [2] | 1350 | [2] |
| CAPl | 0.036 |  | 2000 |  |
| CAPo | 0.060 |  | 1500 |  |
| CAPm | 0.036 |  | 2000 |  |
| cPT | 0.053 |  | 6000 |  |
| mPT | 0.054 |  | 6000 |  |
| lPT | 0.054 |  | 6000 |  |
| mPFL | 0.057 | [12] | 1965 | [13] |
| lPFL | 0.032 |  | 1159 |  |

**
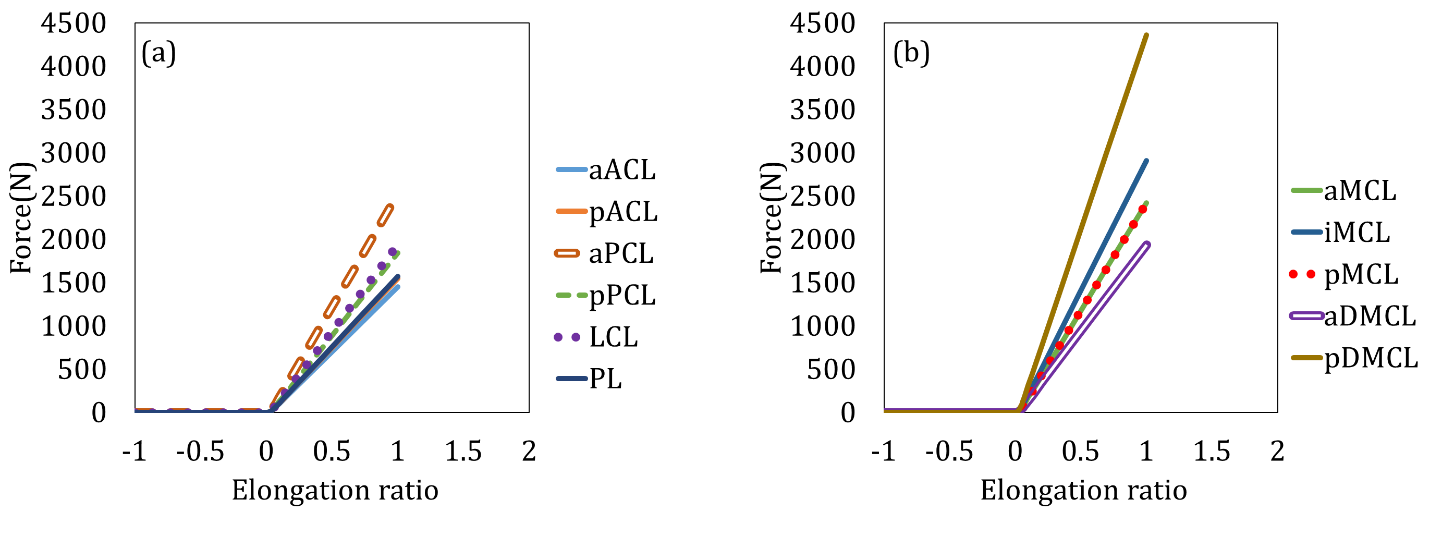

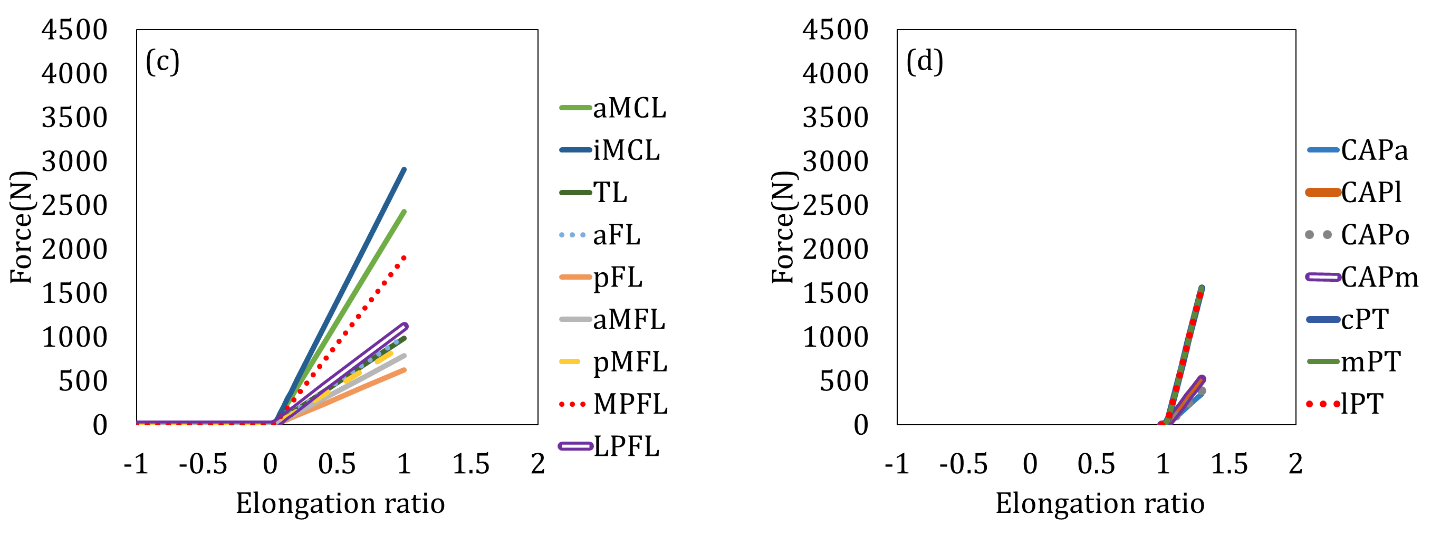
**Figure S2. Force elongation Characterstics of all connecting tissues

Figure S3. Passive strain behavior of PFL, FL and TL

REFERENCE

[1] E. M. Arnold, S. R. Ward, R. L. Lieber, and S. L. Delp, “A model of the lower limb for analysis of human movement,” *Ann. Biomed. Eng.*, vol. 38, no. 2, pp. 269–279, 2010.

[2] A. Schmitz and D. Piovesan, “Development of an Open-Source, Discrete Element Knee Model,” *IEEE Trans. Biomed. Eng.*, vol. 63, no. 10, pp. 2056–2067, Oct. 2016.

[3] S. L. Delp *et al.*, “OpenSim: Open source to create and analyze dynamic simulations of movement,” *IEEE Trans. Biomed. Eng.*, vol. 54, no. 11, pp. 1940–1950, 2007.

[4] T. P. Exarchos, A. Papadopoulos, and D. I. Fotiadis, *Handbook of research on advanced techniques in diagnostic imaging and biomedical applications*. Medical Information Science Reference, 2009.

[5] A. Schmitz and D. Piovesan, “Development of an open-source cosimulation method of the knee,” in *2016 38th Annual International Conference of the IEEE Engineering in Medicine and Biology Society (EMBC)*, 2016, vol. 2016–Octob, pp. 6034–6037.

[6] M. Kupczynska, K. Barszcz, P. Janczyk, M. Wasowicz, and N. Czubaj, “Morphology of the transverse ligament of the atlas and the alar ligaments in the silver fox (Vulpes vulpes var).,” *BMC Vet. Res.*, vol. 9, no. 1, p. 64, 2013.

[7] J. M. Paci, M. G. Scuderi, F. W. Werner, L. G. Sutton, P. F. Rosenbaum, and J. P. Cannizzaro, “Knee Medial Compartment Contact Pressure Increases with Release of the Type I Anterior Intermeniscal Ligament,” *Am. J. Sports Med.*, vol. 37, no. 7, pp. 1412–1416, 2009.

[8] J. E. McDermott, P. E. Scranton, and J. V. Rogers, “Variations in Fibular Position, Talar Length, and Anterior Talofibular Ligament Length,” *Foot Ankle Int.*, vol. 25, no. 9, pp. 625–629, Sep. 2004.

[9] D. C. Marchetti *et al.*, “The Proximal Tibiofibular Joint: A Biomechanical Analysis of the Anterior and Posterior Ligamentous Complexes,” *Am. J. Sports Med.*, vol. 45, no. 8, pp. 1888–1892, 2017.

[10] a R. Poynton, S. M. Javadpour, P. J. Finegan, and M. O’Brien, “The meniscofemoral ligaments of the knee,” *J Bone Jt. Surg Br*, vol. 79, no. 2, pp. 327–330, 1997.

[11] C. M. Gupte, A. Smith, N. Jamieson, A. M. J. Bull, R. D. W. Thomas, and A. A. Amis, “Meniscofemoral ligaments - Structural and material properties,” *J. Biomech.*, vol. 35, no. 12, pp. 1623–1629, 2002.

[12] S. Capkin, G. Zeybek, I. Ergur, C. Kosay, and A. Kiray, “An anatomic study of the lateral patellofemoral ligament,” *Acta Orthop. Traumatol. Turc.*, vol. 51, no. 1, pp. 73–76, 2017.

[13] H. U. Stäubli, L. Schatzmann, P. Brunner, L. Rincón, and L.-P. Nolte, “Mechanical Tensile Properties of the Quadriceps Tendon and Patellar Ligament in Young Adults,” *Am. J. Sports Med.*, vol. 27, no. 1, pp. 27–34, 1999.
